# Genetic interaction between *RLM1* and F-box motif encoding gene *SAF1* contributes to stress response in *Saccharomyces cerevisiae*

**DOI:** 10.1186/s41021-021-00218-x

**Published:** 2021-10-09

**Authors:** Meenu Sharma, V. Verma, Narendra K. Bairwa

**Affiliations:** Genome Stability Regulation Lab, School of Biotechnology, Shri Mata Vaishno Devi University, Katra, Jammu & Kashmir 182320 India

**Keywords:** *S. cerevisiae*, F-box motif, E-3 ligase, Stress

## Abstract

**Background:**

Stress response is mediated by the transcription of stress-responsive genes. The F-box motif protein Saf1p is involved in SCF-E3 ligase mediated degradation of the adenine deaminase, Aah1p upon nutrient stress. The four transcription regulators, *BUR6, MED6, SPT10, SUA7,* are listed for *SAF1* in the genome database of *Saccharomyces cerevisiae.* Here in this study, we carried out an *in-silico* analysis of gene expression and transcription factor databases to understand the regulation of *SAF1* expression during stress for hypothesis and experimental analysis.

**Result:**

An analysis of the GEO profile database indicated an increase in *SAF1* expression when cells were treated with stress agents such as Clioquinol, Pterostilbene, Gentamicin, Hypoxia, Genotoxic, desiccation, and heat. The increase in expression of *SAF1* during stress conditions correlated positively with the expression of *RLM1,* encoding the Rlm1p transcription factor. The expression of *AAH1* encoding Aah1p, a Saf1p substrate for ubiquitination, appeared to be negatively correlated with the expression of *RLM1* as revealed by an analysis of the Yeastract expression database*.* Based on analysis of expression profile and regulatory association of *SAF1* and *RLM1*, we hypothesized that inactivation of both the genes together may contribute to stress tolerance. The experimental analysis of cellular growth response of cells lacking both *SAF1* and *RLM1* to selected stress agents such as cell wall and osmo-stressors*,* by spot assay indicated stress tolerance phenotype similar to parental strain however sensitivity to genotoxic and microtubule depolymerizing stress agents.

**Conclusions:**

Based on in-*silico* and experimental data we suggest that *SAF1* and *RLM1* both interact genetically in differential response to genotoxic and general stressors.

**Supplementary Information:**

The online version contains supplementary material available at 10.1186/s41021-021-00218-x.

## Introduction

The natural population of eukaryotic cells, mostly microorganisms remains non-dividing state and proliferate upon nutrients availability [1]. The proliferation of *Saccharomyces cerevisiae* cells from in and out of the quiescence phase due to nutrients availability or stress condition remains an active research area for both basic research and biotechnological purpose. Upon nutrient deprivation, yeast cells stop dividing and enter into a stationary phase. This transition leads to glycogen accumulation and reduced cell wall porosity which makes cells resist stressors which increases survival probability. The mutants with cell cycle transition defects  that are not able to enter into the stationary phase showed sensitivity to stress condition and may die due to starvation [2]. The studies on gene expression during entry into stationary phase [3] listed thousands of genes with varied expression profile [4, 5] implying that active transcriptional regulation is necessary for entry or exit from stationary phase. The cell cycle phase transition process is dependent on the ubiquitin-proteasome system [6] and the ubiquitination process is essential for survival during starvation. The SCF^Saf1^-E3 ligase in *S.cerevisiae* recruits Aah1p (adenine deaminase) for ubiquitin-mediated degradation upon nutrient deprivation condition [7]. The adenine deaminase is an important enzyme of purine biosynthesis and it requires for conversion of adenine to hypoxanthine. The *AAH1* expression is down regulated during the transition from proliferative state to quiescence state by Srb10p and Srb11p however regulation at the post-transcriptional level of Aah1p requires SCF^Saf1^-E3 ligase activity. The SCF^Saf1^-E3 ligase also requires for degradation of the unprocessed vacuole and lysosomal proteins [8] and three proteins, serine protease B (*PRB1*), protease C (*PRC1*), and Ybr139w reported to be targets of Saf1p for proteasomal degradation [8]. The expression *SAF1* is regulated by regulatory transcription factors (*BUR6, MED6, SUA7*) reported in a study on chromatin immunoprecipitation-chip analysis upon heat treatment [9] and *SPT10* which encodes, a histone H3 acetylase, reported in a microarray RNA expression-based study [10]. The null mutant of *SAF1* exhibits decreased death rate at elevated temperature [11] and resistance to histone deacetylase inhibitor CG-1521 [12].

The *Saccharomyces cerevisiae RLM1/YPL089C* (resistance to lethality of MKK1P386 over expression) is a MADS-box (Mcm1, Agamous, Deficiens, and serum response factor) family transcription factor which is phosphorylated and activated by MAP-kinase Slt2p [13]. The Rlm1p regulates the expression of genes related to cell wall integrity when cells are treated with calcofluor white and zymolyase [14] indicating its role in stress response in *S.cerevisiae.* Deletion of *RLM1* in *S.cerevisiae* results in resistance to cell wall disrupting agents [15]. *S.cerevisiae RLM1* homologs have been reported from other filamentous fungi such as *Aspergillus nige*r [16], *Aspergillus fumigatus* [17], *and Candida albicans* [18].

The gene expression omnibus (GEO) profile database at the National Centre for Biotechnology Information (NCBI) is a repository of genome-wide gene expression of different model organisms and can be mined for gene expression status using both experiment-centric and gene-centric approach [19, 20]. The gene expression is dependent on the transcriptional network involving the interaction of transcription factors and promoter of genes [21]. The Yeast transcription database YEASTARCT (Yeast Search for Transcriptional Regulators And Consensus Tracking) allows the search of association between the transcription factors and gene expression in a variety of experimental conditions (http://www.yeastract.com). It is a  repository of curated associations between transcription factors and genes in *Saccharomyces cerevisiae* [22]. The Yeast stress expression database, yStreX (http://www.ystrexdb.com/) allows analysis of gene-specific expression patterns during stress. The database has a collection of genome-wide expression data on stress response in *S.cerevisiae* [23].

In the present study, we studied the Gene Expression Omnibus profile database (GEO) for the expression status of *SAF1* during stress conditions, and yeast transcription databases were investigated for  the association of transcription factors with the *SAF1* expression during stress. The regulatory association analysis was carried out to understand, transcriptional regulation of target genes by transcription factors during stress for hypothesis followed by experimental analysis. We report *RLM1* as a transcription factor regulating the expression of *SAF1* during stress through *in-silico* analysis. Further, we hypothesized that ablation of both genes may contribute to stress tolerance. We report that the loss of *SAF1* and *RLM1* together leads to stress resistance in *S. cerevisiae* to selected agents.

## Materials and methods

### Yeast strains and plasmids

*Saccharomyces cerevisiae* strain, BY4741 (*Mata his3Δ1 leu2Δ0 met15Δ0 ura3Δ0)* and *JC2326* (*MAT-ura3, cir0, ura3–167, leu: his, his32 Ty1his3AI-270, Ty1NEO-588, Ty1 (tub: lacs)-146)* and their deletion derivatives used in the study are mentioned in the (Table 1). The list of plasmids used for the generation of deletion marker cassette to replace ORF is mentioned in the (Table 2) and sequences of primers used for PCR are mentioned in (Table 3).

**Table 1:** List of *Saccharomyces cerevisiae* strains used in the study

Strains	Genotype	Source
BY4741	*MATa his3Δ1 leu2Δ0 met15Δ0 ura3Δ0*	
*MRS1*	*saf1∆::HIS3*	This study
*MRS2*	*rlm1∆*:*:KanMX*	This study
*MRS3*	*saf1∆::HIS3, rlm1∆::KanMX*	This study
JC2326	*MAT-ura3, cir0, ura3–167, leu::hisG, his32* *Ty1his3AI-270, Ty1NEO-588, Ty1 (tyb::lacz)-146*	Prof. M. Joan Curcio USA
*MRS4*	*saf1∆::KanMX*	This study
*MRS5*	*rlm1∆::KanMX*	This study
*MRS6*	*saf1∆::KanMX, rlm1∆::LEU2*	This study

**Table 2:** List of plasmids used for generating deletion cassette

Plasmids	Deletion module	Selection media
pFA6a- KanMX6	*KanMX6*	YPD + G418
pFA6a- His3MX6	*HIS3MX6*	SD/ His –

**Table 3:** List of primers sequences used for amplification of deletion cassette

Gene Name	Primer code	Sequence
*SAF1*	SAF1F	5’CCAAAGGATATACTCTCAATTATAAATGA
		AAGCACATCCGGATCCCCGGGTTAATTAA-3’
	SAF1R	5’ACGGAATCCAAAATGCAAAATCGAAATGA
		CACCTAAAAA TGAATTCGAGCTCGTTTAAAC-3’
*RLM1*	RLM1F	5’TAAATATTAAAGTGTCGCAAAACTATACTA
		TAGATACAACCGGATCCCCGGGTTAATTAA-3’
	RLM1R	5’TCTTATGCTTGGAATATTCATACTGGTCAA
		ATTTTTTGGTGAATTCGAGCTCGTTTAAAC-3’
*RLM1*	RLM1FL	5’TAAATATTAAAGTGTCGCAAAACTATACTA
		TAGATACAACCCAACTGTGGGAATACTCAG-3
	RLM1RL	5’TCTTATGCTTGGAATATTCATACTGGTCAA
		ATTTTTTGGTTTGGCCCGAAATTCCCCTAC-3

### Gene expression omnibus profile database (GEO) search for gene expression status

The GEO profile database at (https://www.ncbi.nlm.nih.gov/geoprofiles/) was searched gene-specific keyword (*SAF1*) to study the expression profiles during stress in *S. cerevisiae*.

### Transcriptional regulation databases (YEASTRACT) search for regulators of gene transcription

For the search of transcriptional regulators of a gene in *Saccharomyces cerevisiae*, the database at (http://www.yeastract.com) was searched for global transcription factors during normal and stress conditions for *SAF1* and *AAH1,* further analysis for regulatory association of *SAF1* and *AAH1* with common transcription factors (TF) was carried out during stress condition.

### Yeast stress expression database (yStreX) analysis

The Yeast stress expression database (http://www.ystrexdb.com/) was analyzed for the expression status of *SAF1, AAH1*, and *RLM1* by using an advanced search tool at default cut-off (log2 Fold Change > 1.5) and (adjusted *p*-value at < 0.05) under all experimental classes. The results on the expression status of genes included the statistical values and biological features for specific conditions.

### Growth assay

To compare the growth rate between WT (BY4741) and its deletion derivatives in YPD broth and solid media standard protocols were  adopted. For growth kinetics, a single colony of each strain was inoculated in 25 ml YPD broth and grown overnight at 30 °C. The next day each culture was  diluted to 0.05 optical density (OD) at 600 nm and allowed to grow up to 14 h. The OD after every 2 h for each culture was measured using TOSHVIN UV-800 SHIMADZU spectrophotometer. The experiment was carried out in triplicates and  the average OD was plotted against each time point to compare the growth rate. To compare the growth on solid media each strain was streaked on YPD + agar plates followed by incubation at 30 °C for 2–3 days.

### Microscopy

To compare the morphological features between WT and deletion derivative yeast strains, phase contrast microscopy was carried out on log-phase grown cultures in YPD medium at 30 °C using Leica DM3000 microscope at × 100 magnification. To study the chitin distribution in the WT and mutant strains Calcofluor white staining method was adopted. Briefly, WT and mutant strain were grown overnight at 30 °C and the next day a small aliquot was inoculated in fresh YPD medium in 1:10 ratio for growth till log phase. The cells were suspended in 100 μl of the solution containing Calcofluor white (50 μg/ml solution) fluorescent dye, after staining cells were observed under × 100 magnification of fluorescence microscope using ultraviolet filters as per instructions mentioned in the manual of Leica DM3000 fluorescence microscope.

To study the nuclear migration defects in WT and its deletion derivatives, assay described [24, 25] was adopted. The number of nuclei per cell in yeast strains was determined with nuclear binding dye (DAPI). Briefly, strains were grown to early log phase (OD_600_ ~ 0.8) at 30 °C. Yeast cells were washed with distilled water and suspended in 1X PBS (Phosphate Buffer Saline). Further, fixation was done by the  addition of 70% ethanol before DAPI staining. Cells were washed with 1X PBS buffer then again centrifuged for 1 min at 2500 rpm. DAPI stain (1 mg/ml stock) to  a final concentration of 2.5 μg/ml was added and incubated for 5 min at room temperature and visualized under a fluorescent microscope with 100 X magnifications. A total of 200 cells were counted and grouped according to  0, 1, 2, and multi nuclei per cell, more than two nuclei per cell indicated the nuclear migration defect.

### Spot assay for comparative cellular growth response

For comparative assessment of WT and its deletion derivatives for cellular growth response to stress agents, standard semi-quantitative spot assays were performed. Briefly, wild type (BY4741) and its deletion derivatives strains were grown in the 25 ml YPD (Yeast Extract 1% w/v, Peptone 2% w/v, dextrose 2% w/v) medium overnight at 30 °C. The next day the cultures were diluted and grown in fresh YPD medium for 3–4 h to reach the  log phase (OD600 0.8–1.0). The cultures concentration was adjusted by dilution (equal OD at 600 nm) serially diluted, from each dilution, an aliquot of 3 μl volume was spotted onto YPD agar plates, without stress agents and plated containing Hydroxyurea (HU), Methyl methanesulfonate (MMS), Nocodazole, Benomyl, Calcofluor White, SDS, NaCl, Glycerol, DMSO, H_2_O_2_ as each separate agents. The plates were incubated at 30 °C for 2–3 days and cellular growth of the WT and mutants were recorded. The plates with spot assays were analyzed as per the method mentioned [26] for quantification of the relative yeast growth. The cellular growth of yeast colonies at 10^− 1^, 10^− 2^ and 10^− 3^ dilutions were analyzed for quantification.

### Assay for Ty1 retromobility

To assess the Ty1 retro-mobility in WT and its deletion derivatives assay was adopted mentioned in [27, 28]. The reporter strain *JC2326* was used for the construction of deletion derivatives. The reporter and its deletion derivatives contain a single Ty1 element marked with indicator gene *HIS3AI.* The indicator gene *HIS3* is interrupted by  an artificial intron (AI) in an orientation opposite to the *HIS3* gene transcription. During transcription of marked Ty1 with this arrangement followed by splicing and reverse transcription, generates cDNA with the functional *HIS3* ORF which upon integration into the genome, generates HIS3 positive phototrophs. The quantitative measurement of the HIS3 positive colonies was considered as  the frequency of retromobility. To measure the Ty1 retrotransposition frequency in the WT (*JC2326*; reporter strain) and the deletion derivatives, a single colony of each strains was inoculated into 10 ml YPD broth and grown overnight at 30 °C. The overnight grown cultures were again inoculated in 5 ml YPD at 1:1000 dilutions. The cultures were grown for 144 h at 20 °C. The culture was serially diluted and plated on minimal media (SD/His^−^ plates) followed by incubation at 30 °C for 3–7 days. The three independent replicates of each strain were  used for serial dilution and plated on SD/His^−^ plates. The numbers of HIS+ colonies were counted from each plate and plotted for comparative assessment of Ty1 retrotransposition.

### Statistical analysis

Statistical significance of observations was determined using the paired student t-test. *P*-value is less than 0.05 indicated significance. To compare the statistical significance between groups and within groups one-way ANOVA and posthoc Bonferroni analysis was performed.

## Results

### GEO profile database exhibits increased expression o*f SAF1* during stress condition

The analysis of the GEO profile database showed elevated expression of *SAF1* when cells of S.cerevisiae were treated with Clioquinol, Pterostilbene, Gentamicin, hypoxia, genotoxic, desiccation, and heat stress agents. The summary of the results is  mentioned and Table 4 and in the supplementary data file (Figure S1-S7; source GEO profile database NCBI).

**Table 4:** Status of *SAF1 *expression during stress conditions (source: GEO)

Stress Agent	Up regulation of *SAF1* during Stress	GEO Profile ID
Clioquinol		ID: 66726869
Pterostilbene		ID: 52693374
Gentamicin		ID: 46082374
Hypoxia		ID: 57031974
Genotoxic		ID: 11783774
Desiccation		ID: 38619274
Heat Shock		ID: 89011

### *SAF1* expression during stress correlates with expression of *RLM1*

**Fig. 1:**
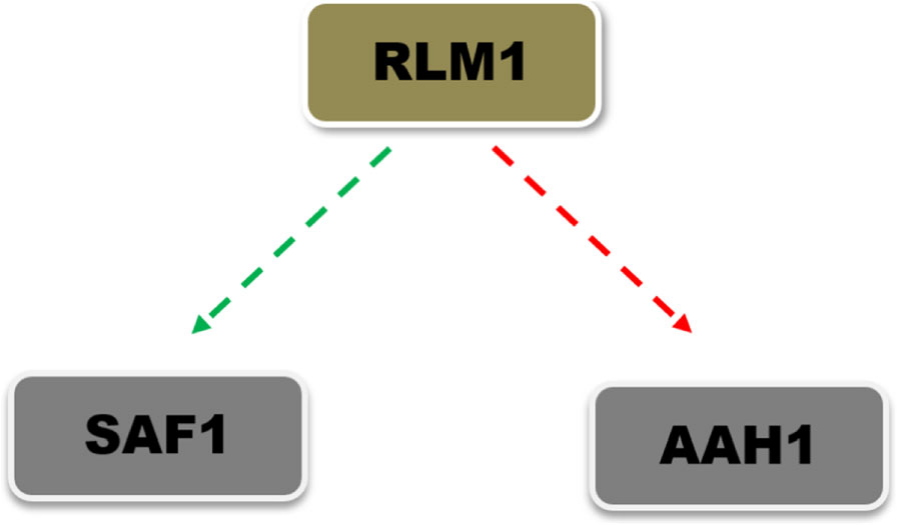
Rlm1p as a transcriptional regulator of *SAF1* and *AAH1* during stress. The Rlm1p acts as a common transcriptional regulator of *SAF1* and *AAH1*, where it associates positively with *SAF1* and negatively with *AAH1*

An *in-silico* analysis using the YEASTRACT database (http://www.yeastract.com) for the association of global transcription factors with *SAF1* during normal and stress conditions revealed a total of three transcription factors i.e. Rlm1p, Spt23p, and Cin5p (Table 5), among three Rlm1p and Cin5p found to be the positive regulator. However six other transcriptions factors i.e. Rpn4p, Msn2p, Msn4p, Aft1p, Rap1p, and Rlm1p showed an association with *AAH1* during stress (Table 5). The Rlm1p was found to be a common transcription regulator of both *SAF1* and *AAH1* (Fig. 1).  Regulatory association analysis showed *RLM1* as a positive regulator of the *SAF1* and negative regulator of the *AAH1*. Furthermore, an analysis of the yeast stress expression database (yStreX) showed a 2-fold elevated expression of *SAF1* and *RLM1* upon stress condition (Table 6). Based on *in-silico* analysis we hypothesized that upon exposure to stress, upregulation of *RLM1* leads to increase expression of *SAF1* and downregulation of *AAH1* resulting in transitioning of cells into the quiescence phase. The Rlm1p was found to be  the common regulator of both *SAF1* and *AAH1.* It is reported that Saf1p during nutrient stress regulates Aah1p activity post-transcriptionally [7]. Further, based on in*-silico* analysis we hypothesized that deletion of *SAF1* and *RLM1* together may contribute to stress tolerance.

**Table 5:** List of global transcription factors regulating *SAF1* and *AAH1* during normal and stress condition (Source: YEASTRACT database)

Name of Gene	Name of TFs	Reference
*SAF1*	**Rlm1p**, Spt23p and Cin5p	(http://www.yeastract.com)
*AAH1*	Rpn4, Msn2, Msn4, Aft1, Rap1 and **Rlm1p**	(http://www.yeastract.com)

**Table 6:** Expression status of *SAF1* and *RLM1* during stress condition (source: yStreX (http://www.ystrexdb.com

Gene	Class	Sub – class	Control	Case	Strain	Log –FC	*P*-value
***RLM1***	Cultivation	Fermentation	2 day	14 days	285	**2.282**	**0**
***SAF1***	Extra stress	Desiccation	Control	100%	BY4743	**2.142**	6.IE-13
***SAF1***	Limiting	Amino acid (AA- Lim)	YPD SD media	15 min and	W303a	**2.224**	5.4E-11
***SAF1***	Media	SD	YPD	15 min and SD media	BY4741	**2.224**	5.4E-11
***SAF1***	Inhibitor	Rapamycin	Control	200 ng/ml	BY4741	**2.057**	9.91154747E-6
***SAF1***	Temperature	37 °C	25 °C	5 min	BY4741	**2.006**	4.88851412E-6
***SAF1***	Inorganic	Hydrogen peroxide	Control	0.4 mM	BY4741	**2.185**	8.132709E-8

### Loss of *SAF1* and *RLM1* together impacts growth rate

**Fig. 2:**
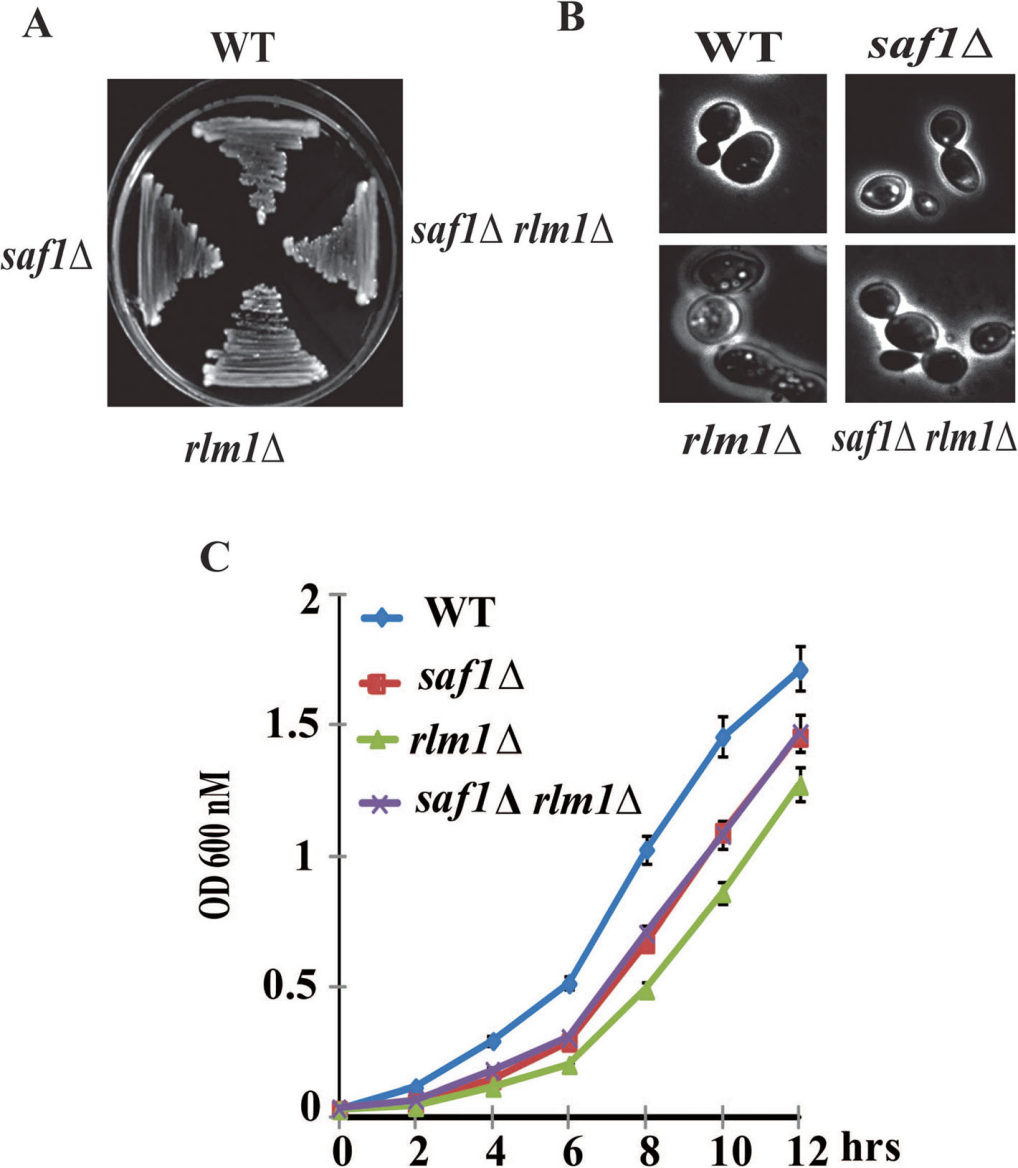
Genetic interaction between *SAF1* and *RLM1* impacts growth rate: Comparative analysis of growth and morphology of cells of WT, *saf1∆, rlm1∆, saf1∆rlm1∆* strain. **A** Comparative growth of streaked strains on YPD agar plates. **B** Representative Phase-contrast images of cells of indicated strains, no major difference concerning morphology observed between WT and mutants **C** Growth kinetics of indicated strains. The *saf1∆, rlm1∆, saf1∆rlm1∆ strains* show a slightly reduced growth rate when compared to the WT strain. The data shown represent the average of three independent experiments. The error bars seen represent the standard deviation for each set of data

Comparative growth assessment of WT, *saf1∆, rlm1∆,* and *saf1∆rlm1∆* yeast strains in liquid and solid rich medium showed a slight reduction in  the growth rate of *saf1∆, rlm1∆, and saf1∆rlm1∆* strains compared to WT (Fig. 2A, C). The analysis of comparative morphological features of cells of WT and mutants strains showed no distinguishable differences (Fig. 2B).

### Loss of *SAF1* and *RLM1* together results in tolerance to CFW and SDS stress

**Fig. 3:**
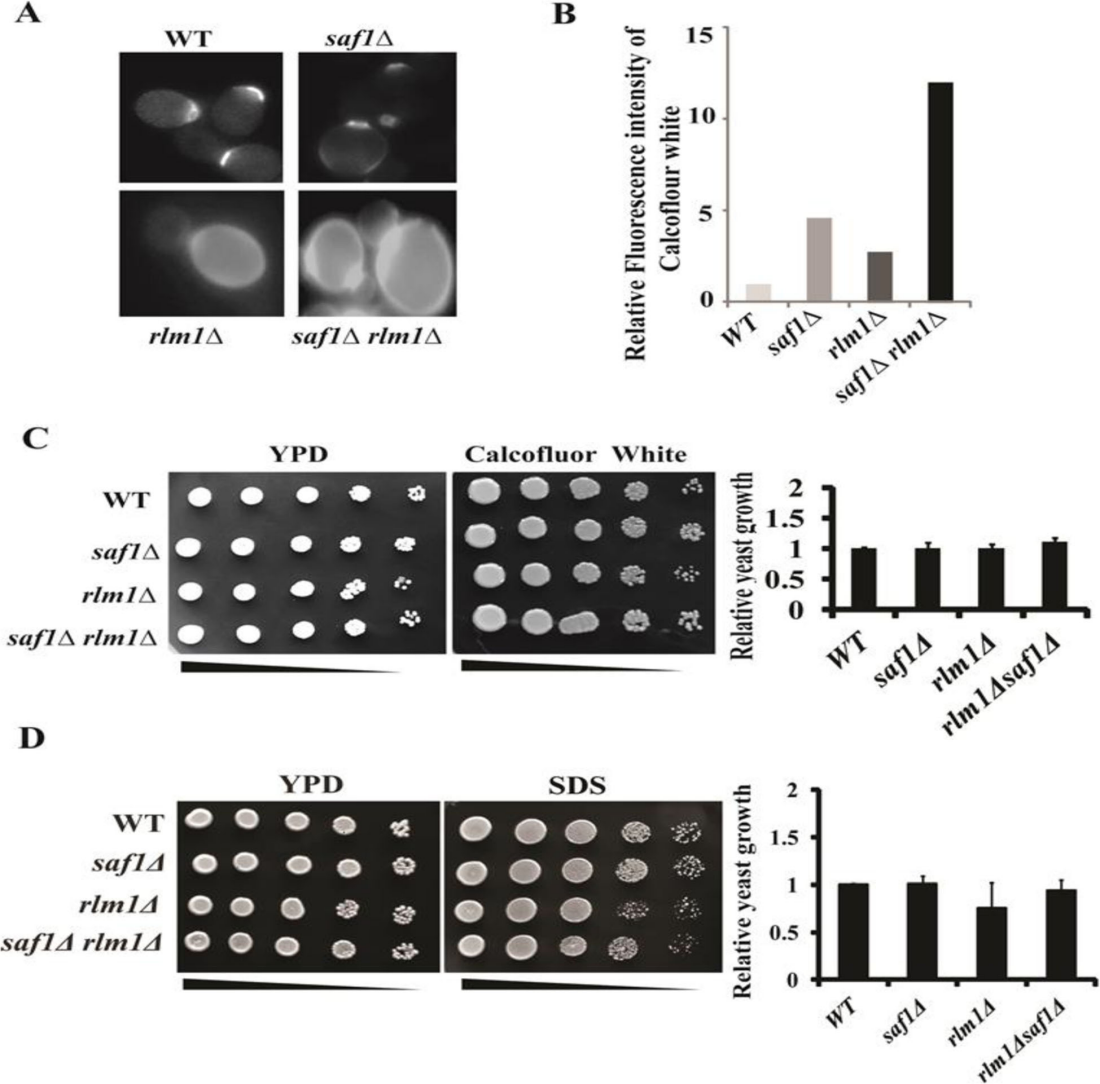
Genetic interaction between *SAF1* and *RLM1* impacts chitin distribution and cellular growth response to cell wall stressors. **A** Representative Fluorescence images of Calcofluor white, specific to chitin in the cell wall, stained cells of WT, *saf1∆, rlm1∆, saf1∆rlm1∆* strains. The cells of *rlm1∆* and *saf1∆rlm1∆* strains showed the altered chitin distribution in the cell wall when compared to cells of WT and *saf1∆* strain. **B** Relative fluorescence intensity of the Calcofluor white stained cells of WT and mutant strains. The *saf1∆rlm1∆* strain showed 10-fold increases in the intensity of fluorescence signal in comparison to WT cells. **C** Images of semi- quantitative spot assay for comparative analysis of cellular growth response of cells in presence of 30 μg/ml Calcofluor white and 0.0075% SDS of WT, *saf1∆, rlm1∆,* and *saf1∆rlm1∆* strains. The cells lacking *SAF1* and *RLM1* together show tolerance to CFW compared to WT, however slight sensitivity to SDS in comparison to WT. The cells lacking *RLM1* show sensitivity to SDS compared to WT cells

Calcofluor white (CFW) is a fluorochrome stain, which binds to chitin, and perturbs the cell wall whereas SDS is a membrane disrupting agent. The cells lacking both *SAF1* and *RLM1* showed altered chitin distribution in comparison to WT and s*af1∆* (Fig. 3A) when stained with CFW. The quantification of relative fluorescence intensity of CFW fluorescence in the strains after staining indicated the elevated level of fluorescence in the cells lacking both *SAF1* and *RLM1 (*Fig. 3B). The spot assay on comparative cellular growth response to CFW (30 μg/ml) and SDS (0.0075%) also showed stress tolerance by cells lacking both *SAF1* and *RLM1* when compared with WT and *rlm1∆* (Fig. 3C, D). Further, the quantification of  the relative growth of yeast strains on plates with stress agents showed a lack of sensitivity by the cells lacking both *SAF1* and *RLM1*, when compared with the WT; however cells lacking only, *RLM1* showed reduced growth when spotted on plates containing SDS as stress agent.

### Loss of *SAF1* and *RLM1* together results in tolerance to oxidative and osmotic stress

**Fig. 4:**
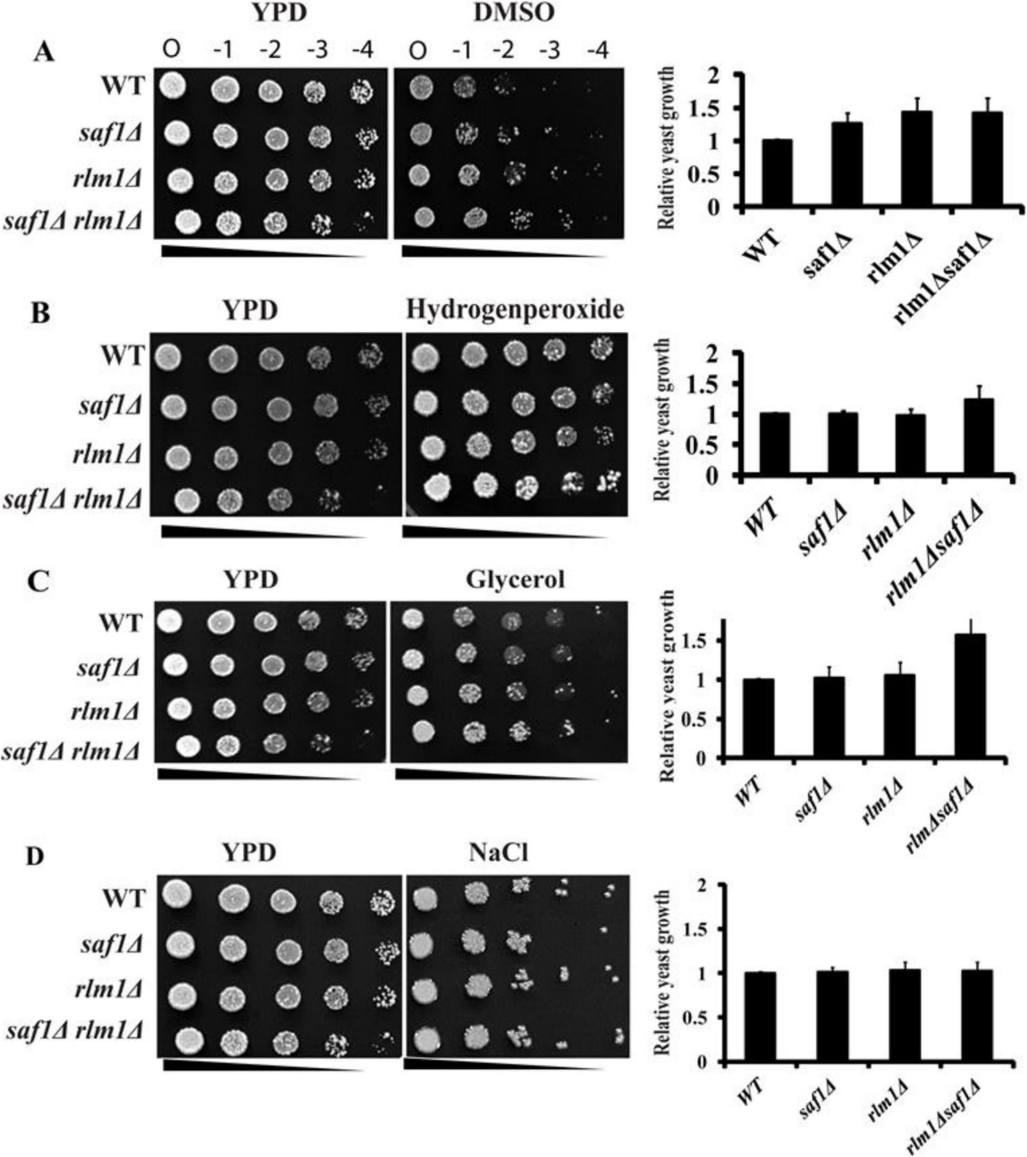
Genetic interaction between *SAF1* and *RLM1* contributes to the cellular growth response to oxidative (DMSO and H_2_O_2)_ and Osmotic (Glycerol and NaCl) stressors **A**, **B**, **C**, **D**. Comparative cellular growth response of cells of WT, *saf1∆, rlm1∆, and saf1∆rlm1∆* strains in presence of 8% DMSO, 2 mM H_2_O_2,_ 4% glycerol, and 1 M NaCl by spot assay. The cells lacking both *SAF1* and *RLM1* together show tolerance to DMSO, hydrogen peroxide, and glycerol compared to WT cells however the response to NaCl appeared similar to WT cells

The hydrogen peroxide (H_2_O_2_) and Dimethyl sulfoxide (DMSO) both are oxidative stress agents [29–31]. The cells lacking both *SAF1* and *RLM1* showed tolerance to 2 mM H_2_O_2_, 8% DMSO (Fig. 4A, B), and 4% of glycerol (Fig. 4C) in the spot assay when compared to WT and *saf11∆* strains The high concentration of external glycerol and NaCl leads to osmotic stress. However, mutants strains grew similar to WT when 1 M NaCl was used as a stress agent (Fig. 4D). This suggests that cells of *saf1∆rlm1∆* strain tolerate osmotic stress mediated by glycerol and are unaffected by  salt stress.

### Loss of *SAF1* and *RLM1* together results in sensitivity to genotoxic stressors

**Fig. 5:**
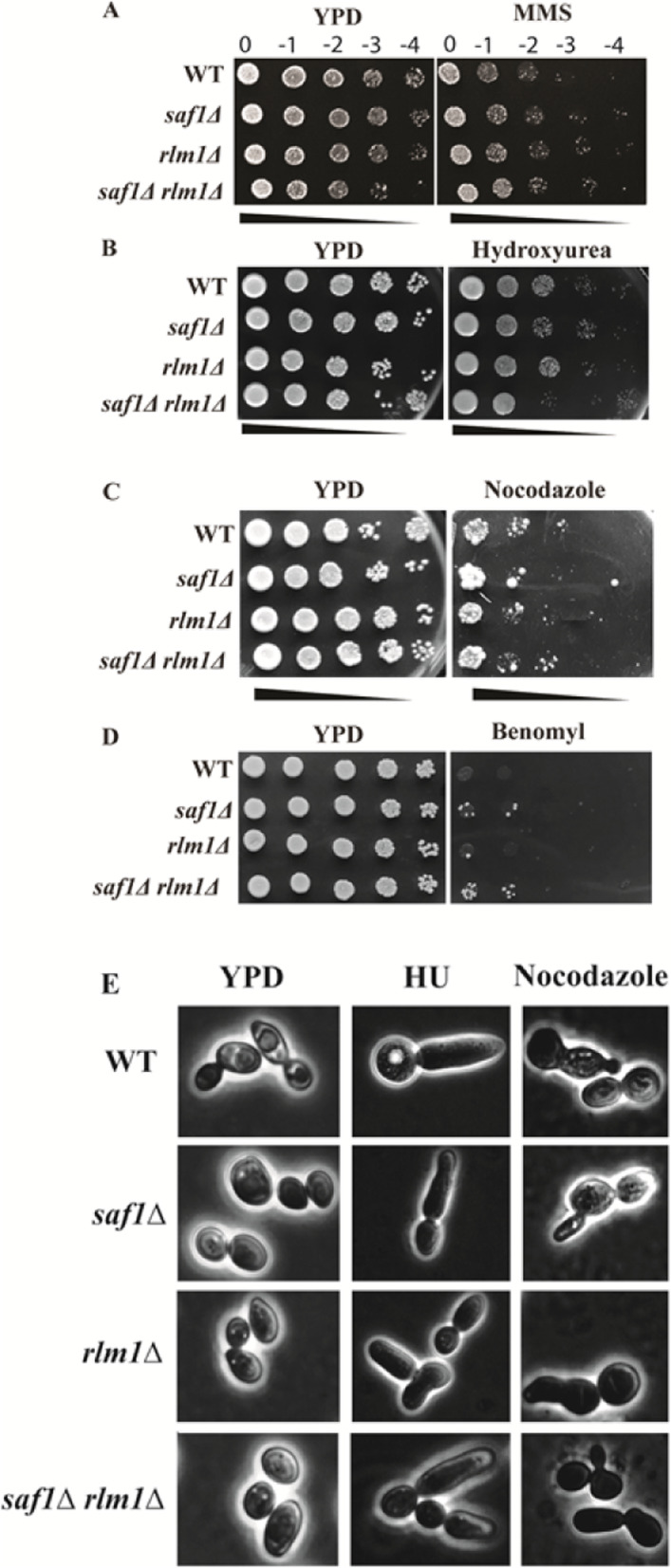
*SAF1* and *RLM1* both require cellular growth response to genotoxic and microtubule depolymerizing agents. **A**, **B**, **C**, **D** Comparative cellular growth response of cells of WT, *saf1∆, rlm1∆, saf1∆rlm1∆* strains in presence of 0.035% MMS, 200 mM HU, 50 μg/ml Nocodazole and 100 μg/ml benomyl by spot assay. The cells lacking *SAF1* and *RLM1* together show sensitivity to HU, Nocodazole and Benomyl compared to WT however, similar growth profile to WT in case of growth on plates with MMS. **E** Comparative morphology of cells of WT, *saf1∆, rlm1∆, and saf1∆rlm1∆* strains grown on YPD + agar media alone and media with HU and Nocodazole stress agents. Cells grown on genotoxic agents show altered cell division

DNA alkylating agent, methyl methanesulfonate (MMS) elicits DNA damage in cells, which results in activation of DNA damage repair pathways [32]. The replication checkpoint inhibitor Hydroxyurea causes DNA replication defects by inhibiting the activity of ribonucleotide reductase (RNR) activity [33]. The cells lacking both *SAF1* and *RLM1* showed growth similar to WT in presence of 0.035% MMS, however, showed sensitivity to 200 mM HU in the spot assay when compared with WT (Fig. 5B, C). The cells lacking both *SAF1* and *RLM1* also showed the sensitive phenotype to microtubule depolymerizing agents, benomyl (100 μg/ml) and Nocodazole (50 μg/ml) in the spot assay (Fig. 5C, D) including WT. All the strains showed the characteristics of elongated buds and altered morphological features when cells were exposed to Hydroxyurea and Nocodazole (Fig. 5E) when compared to the morphology of cells grown on YPD+ agar media alone.

### Loss of *SAF1 and RLM1* together results in increased multi-nuclei phenotype and Ty1 retro-transposition

**Fig. 6:**
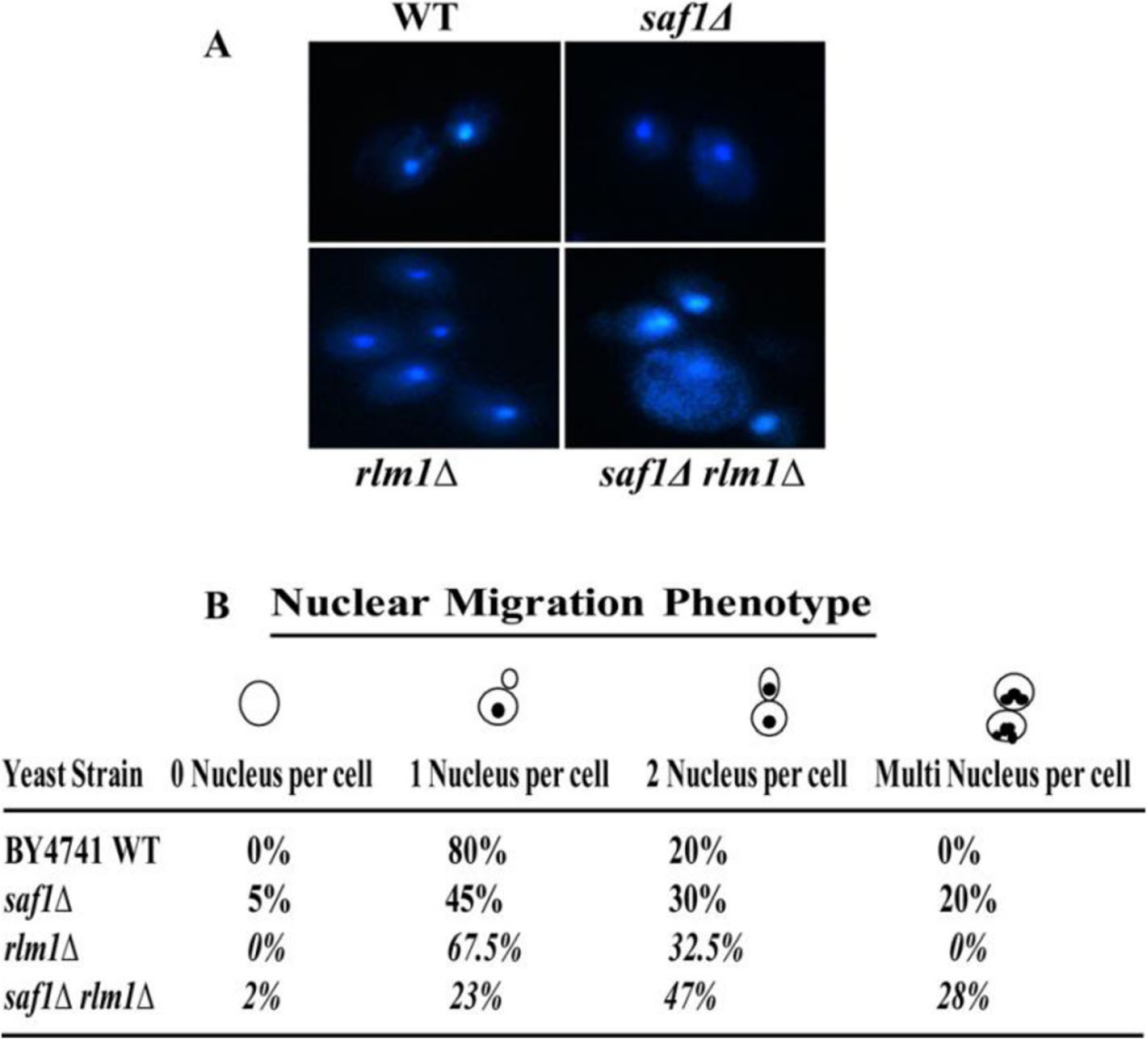
Loss of *SAF1* and *RLM1* together impacts nuclear migration in *S.cerevisiae*. **A** Representative DAPI (4′,6-diamidino-2-phenylindole) stained fluorescent images of log-phase grown cells of WT, *saf1∆, rlm1∆, saf1∆rlm1∆* strains showing the status of Nuclear DNA. **B** Table showing the percent cells with 0, 1, 2, and multi-nuclei feature from the count of 200 cells for each strain, more than two nuclei indicate the nuclear migration defect. The 28% of cells of *saf1∆rlm1∆* strain showed multi-nuclei phenotype compared to WT strains

**Fig. 7:**
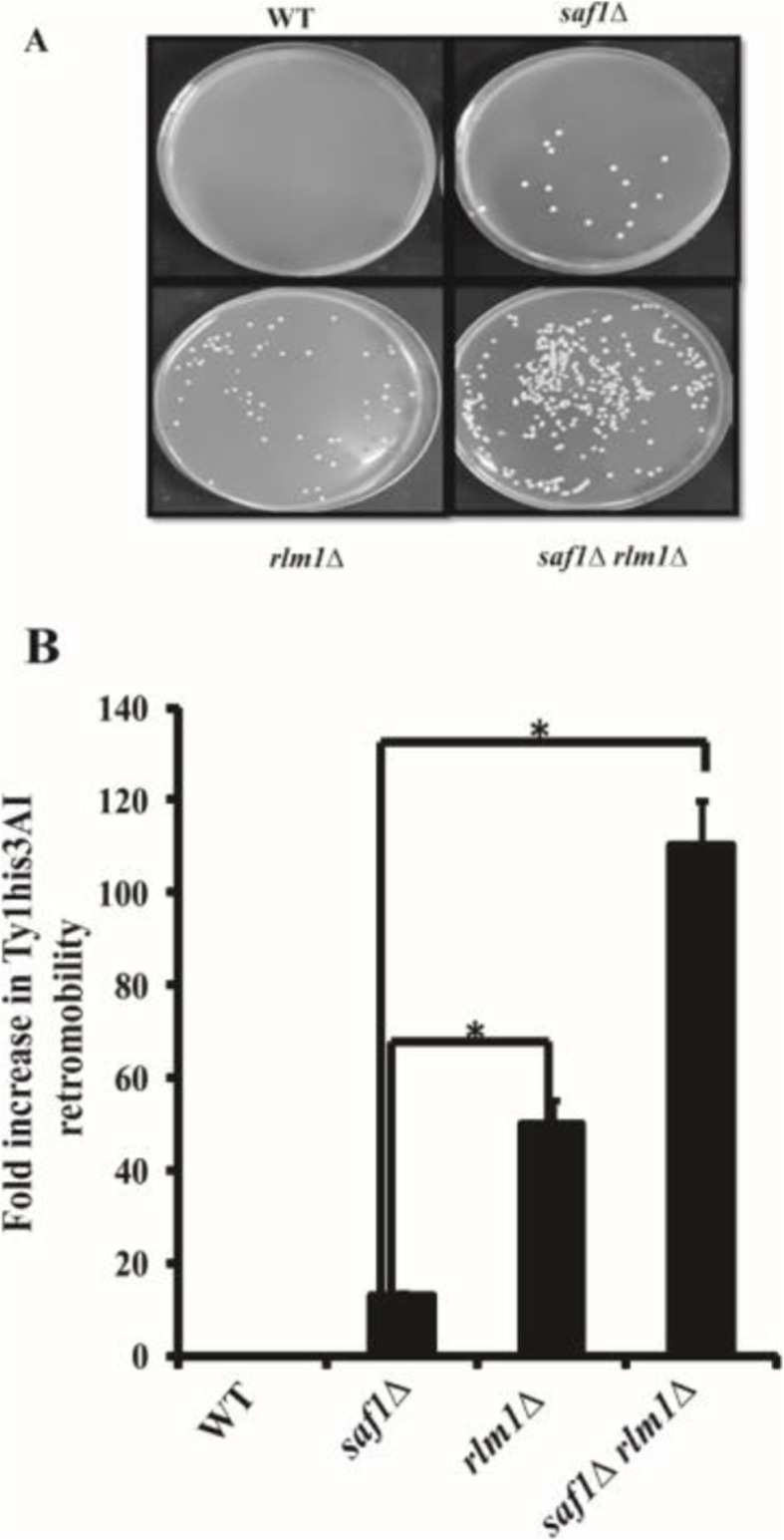
Loss of *SAF1* and *RLM1* together results in the elevated level of retrotransposition of the Ty1 element. **A** Images of plates showing the Ty1 transposition induced HIS^+^ protrophs formation on SD + agar plate lacking Histidine. **B** Bar diagram showing the relative numbers of HIS^+^ prototrophs generated through the retro-transposition activity of the marked *Ty1his3AI* element in each strain. The data shown represent the average of three independent experiments. The significance of retro-transposition event was determined using a two tailed t- test and P–value less than 0.05 was considered as significant. To compare the differences between the groups the one-way ANOVA and posthoc Bonferroni analysis was performed. The one-way ANOVA analysis showed the *p-*value (4.1E-08) between the groups whereas posthoc Bonferroni analysis results showed the p-value as (0.0042) when *saf1Δ* versus *rlm1Δ* and p-value as (0.0031) when *saf1Δ* versus *rlm1Δ saf1Δ* was compared

The sensitivity to genotoxic stress of the cells with loss of both *SAF1* and *RLM1* prompted us to study the nuclear status in the cells using DAPI staining followed by fluorescence microscopy The DAPI stained cells showed compact nuclei as single dot and multi-nuclei feature (Fig. 6A). The quantification of the single and multi-nuclei features in cells of WT, *saf1∆, rlm1∆,* and *saf1∆rlm1∆* (Fig. 6B) showed 28% of cells of *saf1∆rlm1∆* strain with multi nuclei phenotype compared to WT cells which mostly showed compact nuclei. It is well established that DNA replication stress and DNA damage both lead to an increase in Ty1 retromobility in *S. cerevisiae* [34]. The cells lacking both *SAF1* and *RLM1* in JC2326, Ty1 reporter strain background showed ~ 120-fold increase in Ty1retromobility in comparison to WT (Fig. 7A, B) whereas *rlm1∆* strain showed nearly ~ 40-fold increase in the marked Ty1 retro-mobility. The results on multi-nuclei features and Ty1 retro-mobility suggest the role of both the *SAF1* and *RLM1* in genome maintenance through an unknown mechanism  that  needs to be investigated in future studies.

## Discussion

In this study, we report the genetic interaction between transcription factor encoding *RLM1* and F-box motif encoding *SAF1* which impacts the growth rate, response to stress agents, nuclear migration, and retrotransposition of Ty1 element in *S. cerevisiae*. The increased expression of *SAF1,* supported by results of *in-silico* analysis, during a variety of stress conditions suggests the crucial role of *SAF1* in response to stress by *S. cerevisiae.* It is reported earlier that Saf1p is needed for induction of quiescence state in actively dividing cells upon nutrients deprivation stress [35]*.* The Saf1p as SCF^Saf1p^ E-3 ligase complex recruits Aah1p for ubiquitination and subsequent degradation by 26S proteasome during nutrients stress [7, 35]. This implies that the steady level of Aah1p is needed for  the active dividing state of *S.cerevisiae* cells. The observations with reference to *SAF1* expression by *in-silico* analysis prompted us to search for transcription factors  that  associate with the increased expression of *SAF1* during stress. The search for transcription factors using  the YEASTRACT database suggested Rlm1p as a novel transcription factor for *SAF1* during stress. Further, the analysis of regulatory association search between *SAF1, AAH1* and *RLM1* showed *RLM1* as a positive regulator of *SAF1* and negative regulator of *AAH1* genes expression which is supported by the fact that both *SAF1* and *RLM1* expression is increased during stress conditions whereas the expression of *AAH1* is reduced. Further, these observations support the *AAH1* gene model for studying phase transition from proliferative state to quiescence phase upon nutrition stress. Moreover, it is expected that during stress conditions when cells enters into  the quiescence phase for survival, regulation of the steady-state level of *AAH1/*Aah1p must be modulated. Based on  an *in-silico* analysis of the expression and transcriptional database, we report that *Rlm1p* as a transcriptional regulator of *SAF1* and *AAH1* during stress. In *S.cerevisiae* cells during stress, the adoptive transcriptional cascade is initiated in response to stress which is dependent on the Rlm1p transcription factor and mitogen-activated protein kinase Slt2 [36]. Rlm1p is crucial for response to oxidative stress [37], cell wall integrity pathway and is auto regulated during cell wall stress [36]. The deletion of *RLM1* leads to impairment of transcriptional response during cell wall stress which impacts the cell wall integrity [36]. The cell wall integrity pathway is important for both entry, maintenance and, exit from the quiescence phase [38]. The Rlm1p homolog in other filamentous fungi regulates carbon metabolism [18], cell wall integrity and virulence [16, 17, 39]. These reports suggest the role of Rlm1p in response to stress through transcriptional regulation of other genes in *S.cerevisiae.*

Further, based on *in-silico* analysis, we hypothesized that ablation of *SAF1* and *RLM1* together may lead to stress tolerance in the cells lacking both *SAF1* and *RLM1*. Our experimental analysis validated the hypothesis, where cells lacking both *SAF1* and *RLM1* exhibits tolerance to cell wall stressors (Calcofluor white and SDS), oxidative stressors (DMSO and hydrogen peroxide), osmo-stressors (Glycerol and NaCl) compared to parental strain however, cells showed sensitivity to genotoxic stressors (HU) and microtubule inhibitors (Nocodazole and Benomyl) suggesting, the role of other alternate pathways constituted *SAF1* and *RLM1* as part in response to genotoxic stress. This is supported by the fact that cells lacking both *SAF1* and *RLM1* together exhibit an elevated level of Ty1 retrotransposition and multi-nuclei phenotype. It must be noted that the lack of *RLM1* and *SAF1* may contribute to the chronic presence of adenine deaminase, Aah1p encoded by *AAH1.*The adenine deaminase enzyme (Aah1p) is crucial for purine metabolism and is involved in the conversion of adenine to hypoxanthine [40] and imbalance in the purine metabolites may have phenotypic consequences [41].

**Fig. 8:**
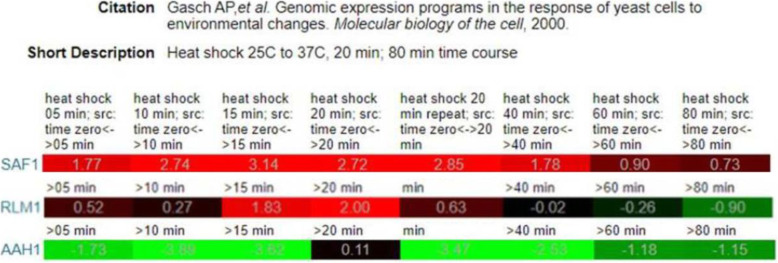
Evidence of sustained expression of *AAH1* during heat stress when expression of *SAF1* and *RLM1* is minimal. Data generated using Serial Pattern of Expression Levels Locator (SPELL Version 2.0.3) database

**Fig. 9:**
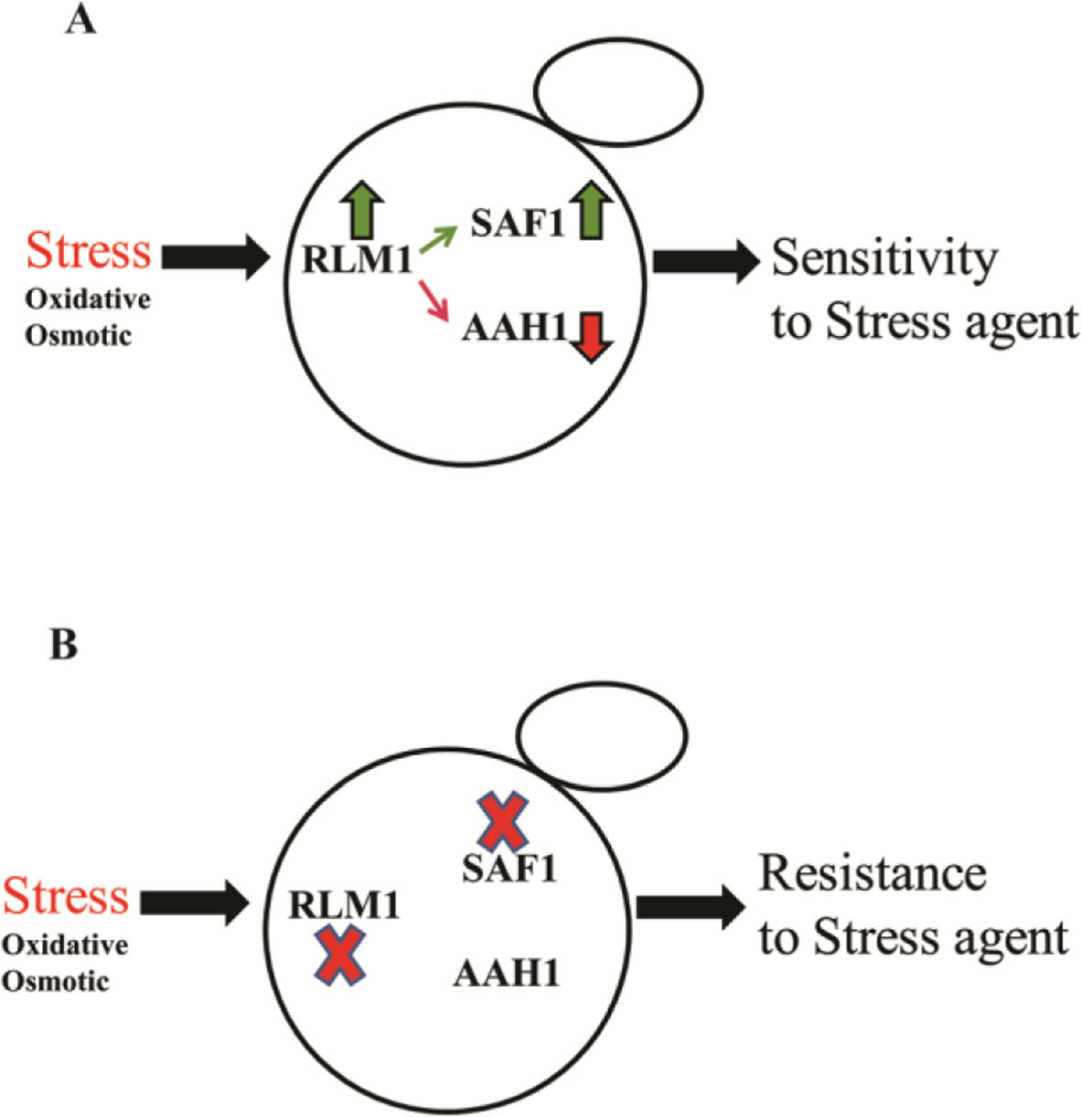
Model indicating the  regulation of stress response, mediated by genetic interaction between *SAF1* and *RLM1* **A**. Stress mediated increased expression of *RLM1* results in decreased transcription of *AAH1 whereas increased transcription of SAF1 that *results in post-transcriptional degradation of Aah1p in *S.cerevisiae,*  that may contribute to sensitivity to stress agents and transition from actively dividing state to quiescence phase **B**. The absence of both *RLM1* and *SAF1* leads to resistance phenotype in presence of a general stressor, that may result due to a steady level of Aah1p in the cells

The analysis of Serial Pattern of Expression Levels Locator (SPELL Version 2.0.3) database [42] showed the sustained increased expression of *AAH1* during heat stress when the expression of *RLM1* and *SAF1* was minimal [4] (Fig. 8). We propose that the stress tolerance phenotype of cells lacking *SAF1* and *RLM1* could be due to sustained expression of *AAH1.* Understand the mechanism of general stress tolerance by cells lacking *SAF1* and *RLM1* requires future studies, however, the  present study suggests a model (Fig. 9) where the continuous presence of *Aah1p* could be one of the reasons for the stress tolerance phenotype of cells lacking *RLM1* transcription factor and F-box motif protein-encoding *SAF1,* as both require for regulation of Aah1p. Based on the *in-silico* data analysis and experimental validation we suggest that *RLM1* is a transcriptional regulator of *SAF1* and *AAH1* during stress.

## Conclusions

Based on in-*silico* and experimental data we suggest that *SAF1* and *RLM1* both interact genetically in differential response to genotoxic and general stressors.

## Supplementary Information

**Additional file 1.** Supplementary Figures from S1 to S7.

## Data Availability

All data generated or analysed during this study are included in this article.

## Abbreviations

DAPI: 4′6-diamidino-2-phenylindole
PBS: Phosphate buffered saline
HU: Hydroxyurea
MMS: Methyl methanesulfonate
DMSO: Dimethyl sulfoxide
SDS: Sodium dodecyl sulfate
NaCl: Sodium chloride
H_2_O_2_: Hydrogen peroxide
His: Histidine
YEASTRACT database: Yeast search for Transcriptional Regulators And Consensus Tracking
GEO: Gene expression omnibus
yStreX: Yeast stress expression database
*SAF1*: SCF associated factor 1
*AAH1*: Adenine Amino Hydrolase
*RLM1*: Resistance to lethality of MKK1P386 overexpression
