# Supplementary material for: Genetic interaction between RLM1 and F-box motif encoding gene SAF1 contributes to stress response in Saccharomyces cerevisiae
Source: Genes Environ. 2021 Oct 9;43:45. doi: 10.1186/s41021-021-00218-x (PMC8501602; doi:10.1186/s41021-021-00218-x)
Supplement: Supplementary file 1 — Additional file 1. Supplementary Figures from S1 to S7. [file 41021_2021_218_MOESM1_ESM.docx]

**Supplementary Data (Sharma M Manuscript): source NCBI (GEO Profile database)**

**Figure S1-S7: *SAF1* overexpressed during stress in the WT cells.** GEO profile of *SAF1* in *S.cerevisiae* cells treated with Clioquinol, Pterostilbene, Gentamicin, Hypoxia condition, Genotoxic Stress, Desiccation, and Heat shock.

**Figure S1.** Gene Expression Omnibus profile status *SAF1* (GDS3751/1776425) in *S. cerevisiae* treated with Clioquinol

**Figure S2.** Gene Expression Omnibus profile of the *SAF1* gene (GDS 3245/7098) in *S. cerevisiae* treated with Pterostilbene**.**

**Figure S3.** Gene Expression Omnibus profile of the *SAF1* gene (GDS 2999/7098) in *S. cerevisiae* treated with Gentamicin

**Figure S4.** Gene Expression Omnibus profile of the *SAF1* gene (GDS 3438/7098) in *S. cerevisiae* during Hypoxia as compared to the normoxia.

**Figure S5.** Gene Expression Omnibus profile of the *SAF1* gene (GDS 1299/7098) in *S. cerevisiae* when treated with genotoxic stress agent

**Figure S6.** Gene Expression Omnibus profile of the *SAF1* gene (GDS 2716/7098) in *S. cerevisiae* when subjected to desiccation

**Figure S7.** Gene Expression Omnibus profile of the *SAF1* gene (GDS 15/3511) in *S. cerevisiae* when subjected to heat shock

| 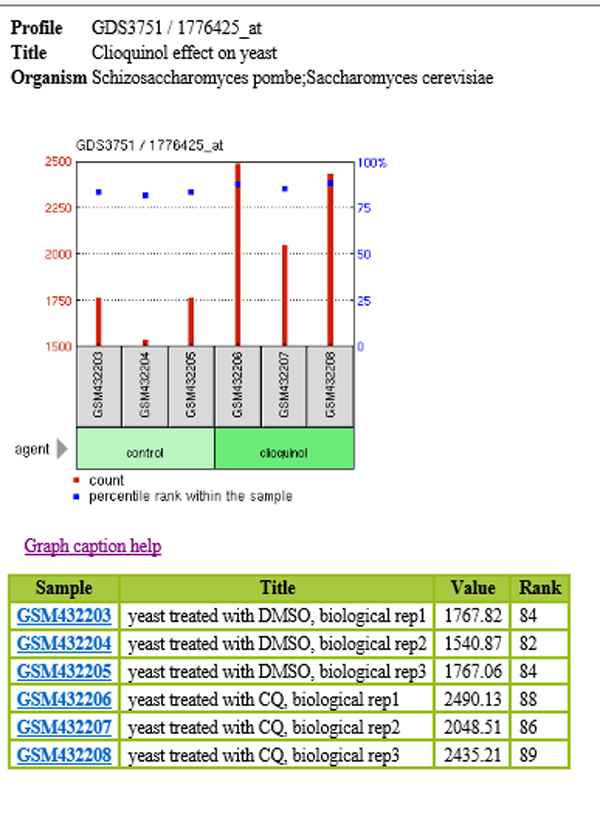  **Figure S1.** Gene Expression Omnibus profile of the *SAF1* gene (GDS3751/1776425) in *S. cerevisiae* treated with Clioquinol. The *SAF1* is upregulated upon treatment with Clioquinol drug. |
| --- |

| 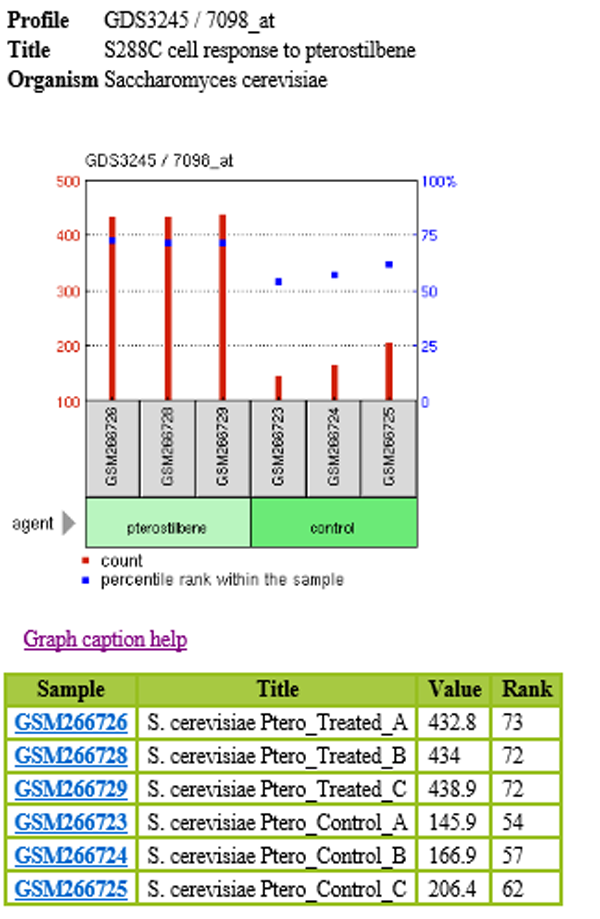  **Figure S2.** Gene Expression Omnibus profile of the *SAF1* gene (GDS 3245/7098) in *S. cerevisiae* treated with Pterostilbene. The *SAF1* is upregulated upon treatment with Pterostilbene drug |
| --- |

| 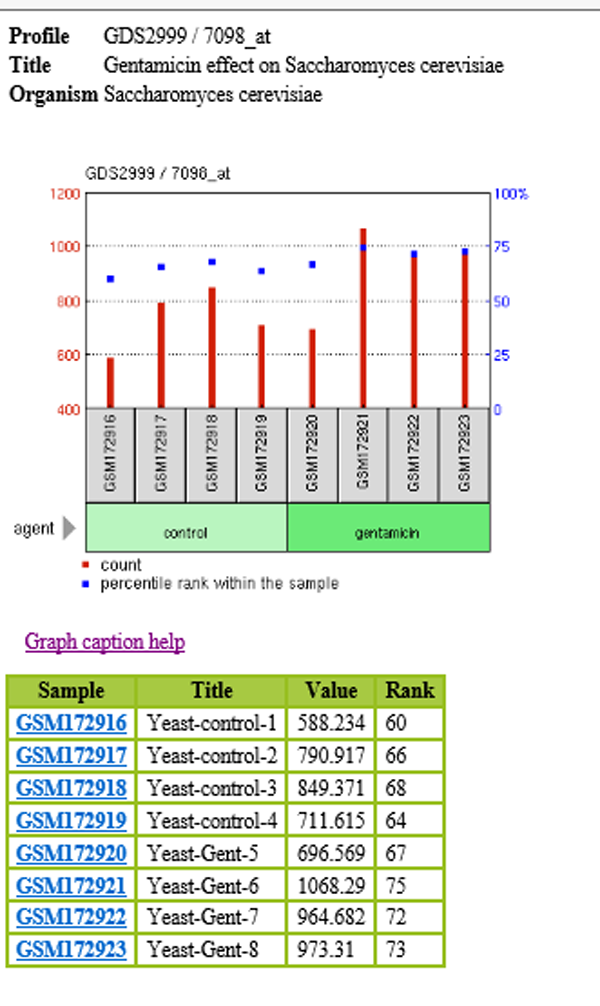  **Figure S3**. Gene Expression Omnibus profile of the *SAF1* gene (GDS 2999/7098) in *S. cerevisiae* treated with Gentamicin. The *SAF1* is upregulated upon treatment with Gentamicin drug. |
| --- |

| **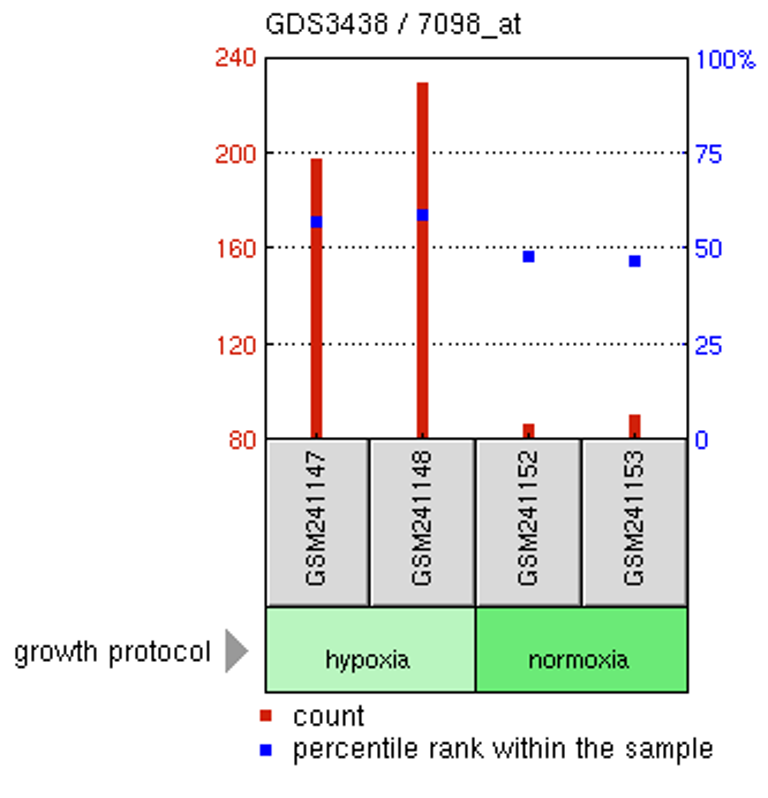**  **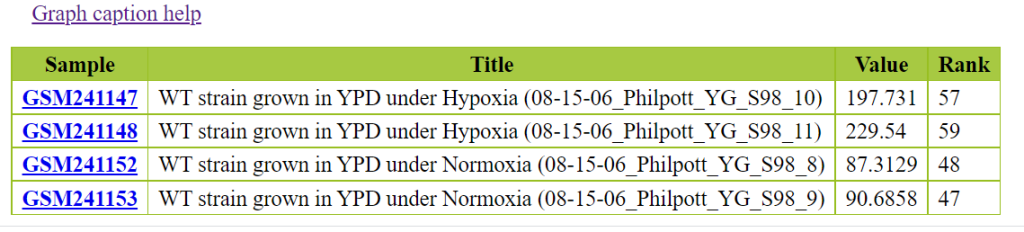**  **Figure S4.** Gene Expression Omnibus profile of the *SAF1* gene (GDS 3438/7098) in *S. cerevisiae* during Hypoxia as compared to the normoxia. The *SAF1* is upregulated upon hypoxia. |
| --- |

| **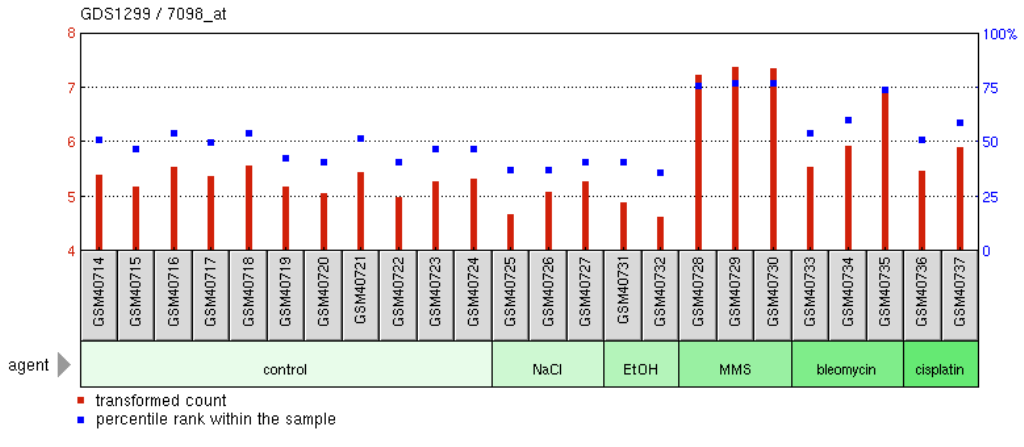**  **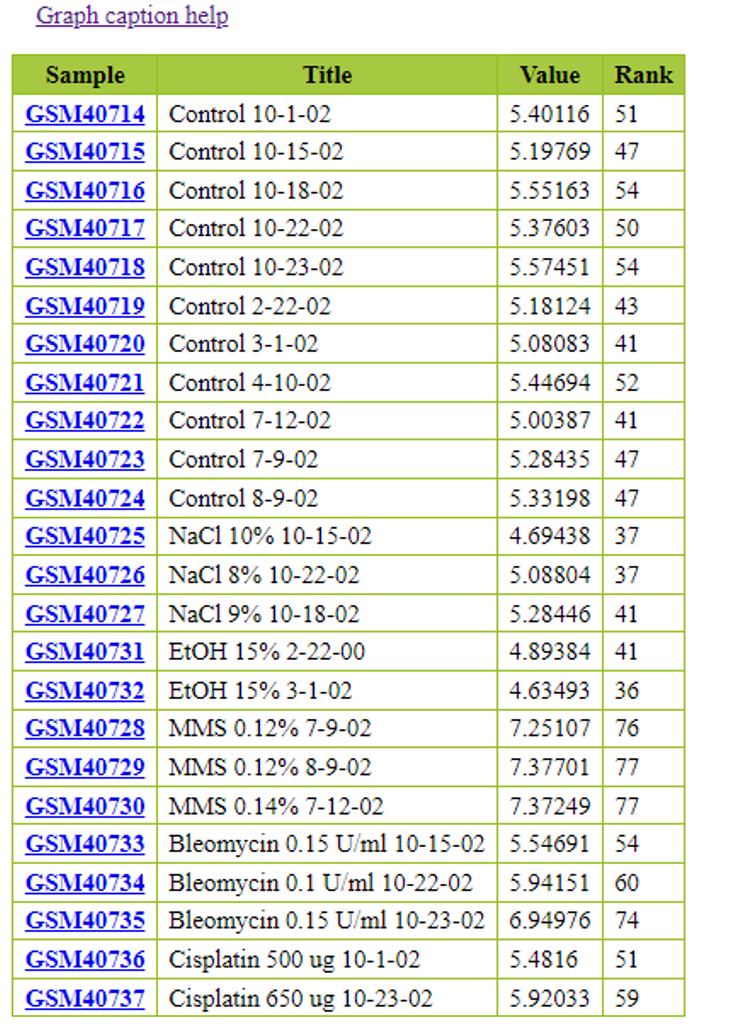**  **Figure S5**. Gene Expression Omnibus profile of the *SAF1* gene (GDS 1299/7098) in *S. cerevisiae* when treated with genotoxic stress agent. The *SAF1* is upregulated upon treatment with genotoxic stress agents such as MMS, Cisplatin. |
| --- |

| **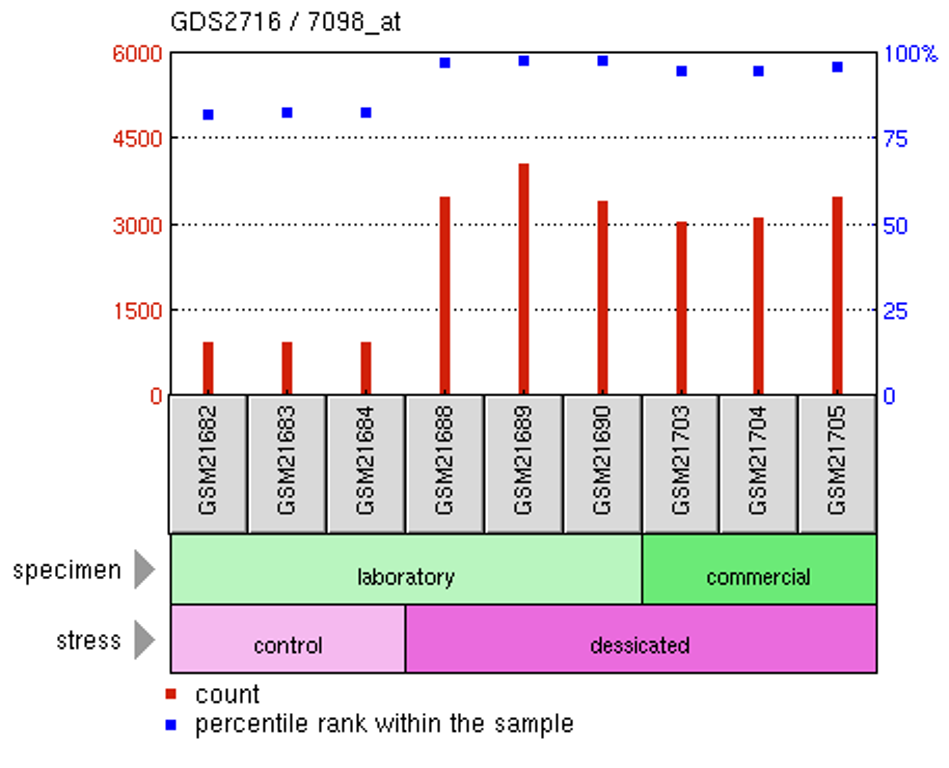**  **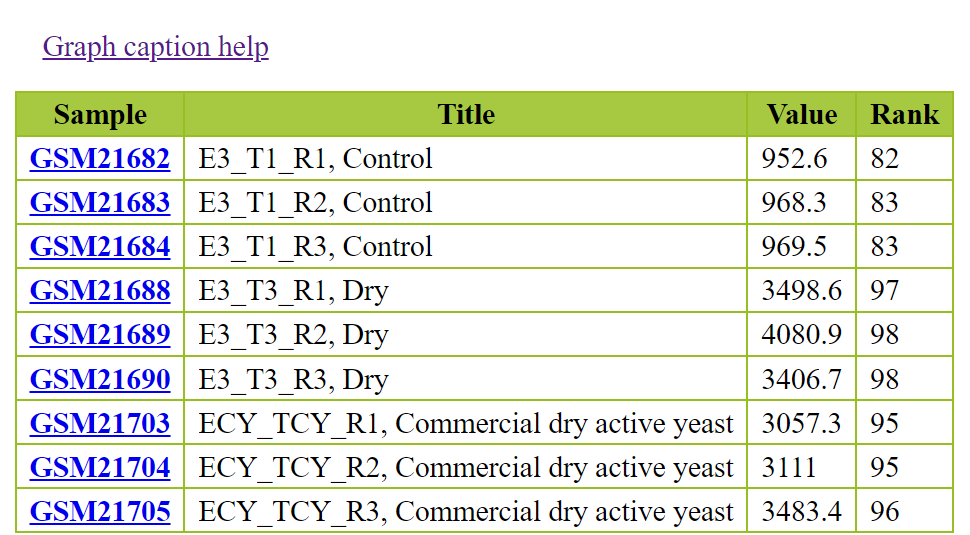**  **Figure S6.** Gene Expression Omnibus profile of the *SAF1* gene (GDS 2716/7098) in *S. cerevisiae* when subjected to desiccation. The *SAF1* is upregulated upon desiccation. |
| --- |

| **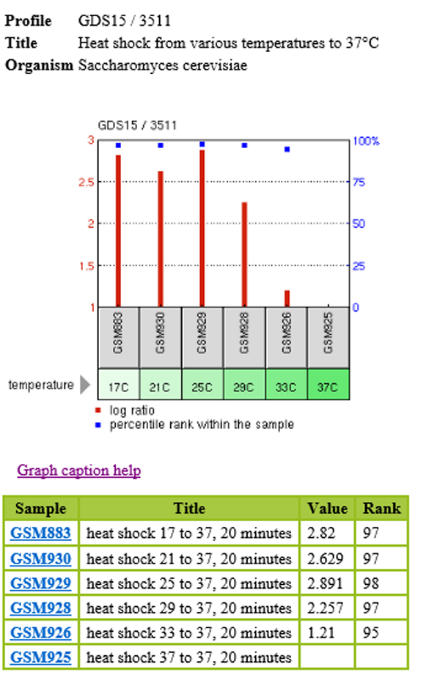**  **Figure S7.** Gene Expression Omnibus profile of the *SAF1* gene (GDS 15/3511) in *S. cerevisiae* when subjected to heat shock. The *SAF1* is upregulated upon heat shock treatment for 20 minutes from 17 to 37 degree Celsius. |
| --- |
